# Digitalization, entrepreneurship and competitiveness: an analysis from 19 European countries

**DOI:** 10.1007/s11846-023-00640-1

**Published:** 2023-03-16

**Authors:** Miguel-Ángel Galindo-Martín, María-Soledad Castaño-Martínez, María-Teresa Méndez-Picazo

**Affiliations:** 1 Facultad de Derecho y Ciencias Sociales, Universidad de Castilla-La Mancha, Ronda de Toledo, s/n, 13071 Ciudad Real, Spain; 2 Facultad de Ciencias Económicas y Empresariales, Ed. Jurídico-Empresarial “Melchor de Macanaz”, Plaza de la Universidad 1, 02071 Albacete, Spain; 3 Facultad de Ciencias Económicas y Empresariales, Universidad Complutense de Madrid, Campus de Somosaguas, 28223 Madrid, Spain

**Keywords:** Competitiveness, Digitalization, Entrepreneurship, Growth, Innovation, L26 (Entrepreneurship), O31 (Innovation and invention)

## Abstract

In light of the economic situation resulting from the COVID-19 pandemic, economists have claimed that an improvement in competitiveness can enhance economic growth. A greater degree of competitiveness allows the relevant actors to engage in entrepreneurial activity in new markets and to create market niches that promote job creation. Among the factors that can stimulate competitiveness, entrepreneurship and digitalization play relevant roles. Digital technologies have generated new business opportunities for entrepreneurs; likewise, digital entrepreneurship allows different entrepreneurs to connect via a platform, thus facilitating access to global markets with growth potential. The fundamental objective of this paper is to study the relationships among digitalization, entrepreneurship and competitiveness in light of the factors that influence the digitalization process. An empirical analysis of 19 European countries is conducted, and fuzzy set qualitative comparative analysis is used to obtain the combinations of economic and social variables that affect competitiveness and entrepreneurship. The results of the empirical analysis show that to stimulate entrepreneurial activity, a country must exhibit an environment that is favourable to digitalization as well as an investment in talent that allows the relevant actors to take advantage of the benefits of digital technologies.

## Introduction

The unstable economic performance, high market volatility and increased uncertainty that have resulted from the COVID-19 pandemic and affected the expectations of economic agents have led, among other issues, to a significant reduction in various countries’ economic growth as well as in the profits and profitability of small and medium enterprises, thereby contributing to a higher level of unemployment.

Faced with the negative effects of this health situation, during postpandemic recovery, countries that want to increase their economic growth must not only consider the new possibilities offered by domestic markets as demand returns to prepandemic levels (Skare and Soriano 2021; Chaturvedi and Karri 2022; Chopra et al. 2022) but also increase the degree of openness of their economies in an attempt to take advantage of market niches provided by foreign markets.

Moreover, the health crisis has accelerated the process of digitalization in European countries digitalization because European economies have been observed to be more vulnerable during this crisis. For this reason, a public aid program was approved to stimulate digital transformation, technological innovation, the digitalization of SMEs and the promotion of talent in European economies with the aim of promoting recovery, competitiveness, economic growth, and job creation in the European Union.

According to EIB (2021), digitalization is essential to increase competitiveness in the European Union and reduce the gap with the United States. Digitalization offers a unique opportunity to improve the global competitiveness of European companies. To close the digital gap, the EU must increase investment and create ecosystems that support innovation and take advantage of the opportunities that disruptive technologies offer to companies. These technologies influence business innovation models and can help companies find new solutions and create value (Åström et al. 2022).

To achieve this objective, it is necessary to promote the competitiveness of these economies, which would prepared companies to enter new markets that may arise more effectively (Lu and Beamish 2001; Zimmerman and Zeitz 2002), as well as to compete with the products made by foreign companies, thus leading ‘to greater growth in the economy and an improvement in living standards’ (Méndez-Picazo et al. 2021; Khanin et al. 2022). Accordingly, the improvement of competitiveness can promote the economic growth of countries (Baldwin 2003; Kordalska and Olczyk 2016; Simionescu 2016; Idris et al. 2018; Alexoaei 2020).

From this perspective, it is necessary to identify the factors that can increase competitiveness. Various factors have been considered: the introduction of government policies that favour business efficiency, the promotion of innovation processes, anti-inflationary policies, infrastructure improvements, etc. However, in addition to these previously identified factors, it is necessary to highlight at least two factors that play an important role in this process and are becoming more relevant as different economies evolve: digitalization (Kraus et al. 2021) and entrepreneurship. Specifically, we focus on entrepreneurship in its initial stages, since in the empirical analysis, we the proportion of the adult population that owns a business that is no more than 3.5 years old as a proxy for entrepreneurship (Reynolds et al. 2000; Bosma et al. 2008).

Entrepreneurship plays an important role in this process (Kraus et al. 2019a, b) since it enables entrepreneurs to take advantage of the opportunities provided by the markets, face the risks that may arise and introduce new ideas and procedures that allow their companies to provide better quality goods and services, thereby offering benefits both to stakeholders and to consumers, who are able to satisfy their needs with better and cheaper products.

In this process, the rapid development of ICTs in countries and their effects on innovations and implementation of those innovations in companies has played a relevant role. Thus, digitalization favours the growth of production in all sectors of the economy and improves the efficiency of industrial processes, thereby identifying investment in the digitalization of productive activities as an essential factor in economic growth (Zaytsev et al. 2020; Dubolazov et al. 2020; Bouncken et al. 2021). Therefore, digitalization could be considered to represent a critical factor in promoting competitiveness and promoting entrepreneurial activity.

The fundamental objective of this paper is to analyse the way in which entrepreneurship contributes to increasing the competitiveness indicators of European countries in light of the digitalization process that European economies are experiencing. The relevance of this analysis lies in, for one, the positive effects of competitiveness on economic growth and the well-being of the countries. A greater degree of competitiveness allows entrepreneurs to engage in entrepreneurial activity in new markets and to create market niches that promote job creation.

## Theoretical framework

The literature on this topic has indicated that an improvement in competitiveness can promote the economic growth of countries (Baldwin 2003; Kordalska and Olczyk 2016; Simionescu 2016; Idris et. 2018; Alexoaei 2020), which can have positive effects on employment and social welfare, especially with respect to promoting the quality and increasing the quantity of goods and services that are offered at a lower price. This fact is especially important in a situation such as the economic suffering resulting from the COVID-19 pandemic, which has caused, among other things, a significant reduction in the levels of growth and employment and, as a consequence, decreased social welfare. Accordingly, the improvement of competitiveness is considered as a factor related to the improvement of the economic situation of such countries with the aim of improving the postpandemic future.

From this perspective, it is important to identify the factors that positively affect competitiveness. Among such factors, innovation has been given special attention (Gavrila et al., 2022; Rubio-Andrés et al. 2022). The introduction of innovation into business activity is fundamentally the result of the aim of obtaining more competitive goods and services, which can allow entrepreneurship to access new markets.

In this sense, the role played by entrepreneurship in this context must be taken into account. It should be noted that innovation, alongside autonomy and opportunities, is one of the three essential elements of entrepreneurship (Lumpkin and Dess 1996; Miller 1983; Stevenson and Jarillo 2007; Lyytinen et al. 2016; Roig‐Tierno et al. 2018; Ferreira et al. 2019; Berman et al. 2022), and according to Schumpeter (1934), it plays an essential role in value creation. With the introduction of such innovations, entrepreneurship leads to the emergence of more competitive goods and services, both in terms of quality and costs.

However, in addition to entrepreneurship activity, digitalization must also be considered (Kraus, Palmer et al. 2019; Kraus et al. 2019a, b). Digitalization has played an important role in the innovative process and therefore in entrepreneurship (Anwar et al. 2022). Digital transformations have provided entrepreneurs with access to new markets, networks, ecosystems, and new customer experiences, thus promoting the emergence of new businesses (Gobble 2018; Fernandes et al. 2022; Guo et al. 2022). This market expansion enables entrepreneurs to satisfy the demands of a greater number of economic agents (Amit and Zott 2001; Gavron et al. 1998; Nambisan 2017; Berger et al. 2021) through three main mechanisms (World Bank 2016, pp. 101–102).

First, digital technologies offer access to labour market information and facilitate the creation of new types of jobs. Accordingly, job seekers can search for the most qualified people to meet their needs and do so more efficiently, thus offering benefits with regard to the labour market (Beckman et al. 2012; Zupic 2014; Trischler and Li-Ying 2022; Bouncken et al. 2020). Second, such technologies have improved the skills of workers, leading to higher productivity (Bowersox et al. 2005; OECD 2017; Chen et al. 2022) and thus a reduction in production costs, making products more competitive, as indicated above. Finally, economies of scale are produced, which in turn stimulate the introduction of new innovations (Deichmann et al. 2016; Trantopoulos et al. 2017; Veiga et al. 2020). In addition, digitalization contributes to the emergence of a more competitive and transparent labour market, which makes it easier to attract, recruit, train, engage and retain the most talented employees (Tanwar 2017; Dorasamy 2021). This situation makes it easier for companies to attract and retain the most talented workers, thereby becoming more competitive in a world that is characterized by digital transformation and accelerated technological change (Nambisan et al. 2019).

With regard to these mechanisms, the importance of training economic agents must be considered, with respect to both accessing new types of work and continuing to generate new innovations that foster competitiveness. Without human capital that can assimilate the digital transformations that are produced by and introduced into the production process, it would be unthinkable to take advantage of the benefits those transformations offer and improve competitiveness. For this reason, it is important to invest in the improvement of human capital and the generation of talent to achieve the aforementioned positive results.

Therefore, the empirical analysis focuses on the effects of these three variables—entrepreneurship, digitalization, and innovation—on competitiveness. This analysis can illustrate the factors that influence entrepreneurship when developing its activity from the perspective of digitalization.

## Empirical analyses

To verify the theoretical aspects thus indicated, an empirical fuzzy set qualitative comparative analysis (fsQCA)is conducted to investigate 19 European countries (Austria, Croatia, Cyprus, Germany, Greece, Ireland, Italy, Latvia, Luxembourg, Netherlands, Norway, Poland, Portugal, Slovakia, Slovenia, Spain, Sweden, Switzerland, and the United Kingdom). The data regarding the variables used in this research are drawn from surveys that collected the information from 2019 until early 2020. To accomplish this task, two equations have been proposed and specified below that allow us to obtain the different causal configurations that explain the paths or patterns that the countries included in the sample follow. It should be noted that fsQCA results are not intended to demonstrate causal relationships but rather to reveal patterns of associations across a series of cases or observations, thus providing support for the existence of such causal relationships (Schneider and Wagemann 2010). Therefore, propositions or research questions are formulated in this type of analysis. Specifically, in our empirical analysis, we try to answer the following research questions:

- Q1. Can innovation affect competitiveness?

As previously indicated, entrepreneurs are the agents who are responsible for introducing innovation into the production process in the attempt to improve their goods and services and attain a better market position. They achieve this goal by improving the quality of those goods or services and/or reducing prices (Kuratko and Audretsch 2009; Lestari et al. 2020; Petrakis et al. 2020). For this reason, a positive relationship between these two variables can be expected.

- Q2. Does the digitalization environment affect entrepreneurship and competitiveness?

Digitalization influences the growth of production in all sectors of the economy and improves the efficiency of industrial processes; accordingly, investment in the digitalization of productive activities represents an essential factor associated with economic growth (Dubolazov et al. 2020; Zaytsev et al. 2020; Endres et al. 2022). In addition, digitalization improves labour productivity and reduces information costs, thereby improving competitiveness (Kraus et al. 2018; Nobanee and Dilshad 2020). Therefore, digitalization is a priority area for the development of innovation processes in economic contexts, as it ensures national and economic competitiveness (Schwab 2019; Autio et al. 2020; Endres et al. 2022; Song et al. 2022).

On the other hand, digitalization has driven the global adoption of new organizational innovations to support the search for business opportunities, such as e-commerce, new forms of financing through venture capital, crowdfunding, coworking spaces, and the expansion of teleworking (Roig‐Tierno et al. 2018; Santoro et al. 2019; Autio et al. 2020; Scuotto et al. 2021); all these changes encourage the emergence of entrepreneurs who try to take advantage of the new business opportunities arising from digital transformation.

- Q3. Does entrepreneurship influence competitiveness?

As previously mentioned, competitiveness is linked to economies' capacity to innovate. Therefore, entrepreneurs positively influence competitiveness because they are the agents who are responsible for introducing innovations into the market at the lowest possible costs in the attempt to obtain a competitive advantage in the market (Petrakis et al. 2020).

Moreover, the inclusion of digital technologies in a business context entails enormous challenges for companies as well as new business opportunities (Peng et al. 2022), as the introduction of these technologies entails the emergence of completely new business models based on revolutionary innovation (Richter et al. 2017). In this sense, the benefits of digital transformation include improvements in productivity, sales, and value creation (Bouncken et al. 2020). These positive effects foster the emergence of new companies with new business models that can increase competitiveness.

- Q4. Does investment in talent encourage entrepreneurship?

Investment in talent or human capital determines the differences in the levels of innovation, entrepreneurship and growth across different geographical areas (Qian 2010); therefore, countries try to attract, retain and invest in talent (IMD 2020). According to Lawson and Samson (2001), for innovation to occur in companies, they must have a highly qualified workforce that cooperates and innovates proactively. Companies therefore need employees who have mastered the digital tools and methods that can enable them to take advantage of the business opportunities offered by digitalization if those companies are to survive and become more competitive (Sivakami 2018; Carlsson 2018; Autio et al. 2020).

- Q5. When there are fewer barriers to digitalization, is entrepreneurship favoured?

Although entrepreneurs have started the process of digitizing their activities due to the opportunities and advantages that digitalization can offer their businesses, they also report certain barriers to digitalization, such as network security problems, uncertainty regarding future digital standards and the lack of financial resources (European Commission 2020; Berman et al. 2022).

### Method and data

fsQCA is a methodology that has been increasingly used in recent years in the social sciences because it allows configural models and determining patterns to be obtained (Ragin 1987, 2000, 2008b; Gannon et al. 2019; Woodside 2018). The main objective of this methodology is to take into account the individual results (or effects) and the patterns (causal configurations) that cause these results (Schneider and Wagemann 2010). This qualitative methodology differs from quantitative techniques in that it aims to reveal the minimum conditions (combinations) that cause a particular result in specific cases (Vis 2012). As a result, asymmetric techniques based on complex theoretical reasoning—such as fuzzy set comparative qualitative analysis (fsQCA)—have recently started to be used to better predict and explain real-world business phenomena using a configurational approach (Kumar et al. 2022).fsQCA allows causal configurations to be found by following a series of steps: (1) identification of all possible causal configurations, (2) analysis of the distribution of cases according to such configurations, (3) detection of sufficient configurations according to a consistency criterion (Ragin 1987), (4) reduction of sufficient configurations by applying counterfactual analysis (Ragin 2008a), and (5) analysis of different combinations of conditions with respect to a problem (Berg-Schlosser et al. 2009). (6) This methodology is also well suited to the task of addressing cases featuring small or medium samples (10 to 50 cases) (Vis 2012; Shipley et al. 2013; Henik 2015; Kraus et al. 2018).

While ordinary and/or generalized least square regressions normally use data from untransformed sources, fsQCA requires a calibration process (Ragin 2008b). According to Ragin (2000), a fuzzy set is “a fine-grained continuous measure that has been carefully calibrated using substantive and theoretical insights relevant to set membership”.

Such data calibration is based on a percentile approach. According to Ragin (2008a), this approach is suitable in cases featuring continuous data. In this calibration process, three points are established (Ragin 2008b): the threshold for full membership (fuzzy score = 0.95), the threshold for full nonmembership (fuzzy score = 0.05), and the crossover point (fuzzy score = 0.5). This final point is defined in terms of the 50th percentile.

Thus, the indicators shown in Table 1 become fuzzy variables expressing the degree of membership, where a value of 1 represents full membership and a value of 0 represents full nonmembership; these terms appear with the notation ‘fs’ to indicate that these variables have been calibrated.

**Table 1:** Definition of variables

Variable	Definition
Competitiveness	fsCOMP: Global Competitiveness Index (Schwab 2019)
Entrepreneurship	fsTEA: Total early-stage entrepreneurial activity (TEA) (GEM 2021) (European Commission 2020) fsINPROC: A new or significantly improved production process or method (European Commission 2020)
Innovation	
Institutions	fsBAR = fuzzyand (fsSEC, fsUNC) fsSEC: Information technology security issues (European Commission 2020) fsUNC: Uncertainty regarding future digital standards (European Commission 2020)
Digitalization system	fsDIGS: The European Index of Digital Entrepreneurship Systems (Autio et al. 2020)
Human capital	fsITAL: Investment & development in talent (IMD 2020)
Venture capital	fsVC: Venture capital total per capita (OECD 2021)

To measure competitiveness (fsCOMP), the Global Competitiveness Index was used. This index integrates the micro- and macroeconomic aspects of competitiveness and evaluates the ability of countries to provide high levels of prosperity to their citizens based on the ability of those countries to productively use the available resources; that is, the index measures the set of institutions, policies and factors that define the levels of sustainable economic prosperity of a country (Sala-i-Martin and Artadi 2004; Schwab 2019).

To measure entrepreneurship, the Total Entrepreneurial Activity (TEA) indicator was used, which refers to the proportion of the adult population (i.e., 18–64 years old) in each country that has some sort of involvement in operating a business that is less than 42 months old. This variable focuses on the number of entrepreneurs whose main motivations include profit, taking advantage of a business opportunity, a need to obtain income, or other reasons (Reynolds et al. 1999; Bosma et al. 2008).

The European Index of Digital Entrepreneurship Systems has been used as an indicator of the digital environment (fsDIGS). This indicator is a composite measure that aims to improve our understanding of and ability to evaluate the digital business ecosystem; that is, it is a measure of both the physical and digital conditions pertaining to stand-up, start-up and scale-up ventures in EU countries and the UK. Simultaneously, this index is integrated by several pillars: culture and informal institutions; formal institutions, regulation and taxation; market conditions; physical infrastructure; human capital; knowledge creation and dissemination; finance; networking and support (Autio et al. 2020).

The Institute for Management Development IMD (2020) World Talent Ranking index captures an economy’s ability to develop and attract talent to strengthen its competitiveness. This index, in turn, is composed of three factors. Our analysis uses “the investment and development factor”, which measures an economy’s ability to foster national talent. Specifically, Investment & Development Factor (fsITAL) is a composite measure that includes the following variables: total public expenditure on education, total public expenditure on education per student, pupil–teacher ratio (primary education), pupil–teacher ratio (secondary education), apprenticeships, employee training, female labour force, and health infrastructure.

To measure “innovation” and “barriers to digitalization”, the information provided by the European Commission in the Flash Eurobarometer 486 is used. This survey provides detailed information regarding SMEs, emerging companies (start-ups), expanding companies (scale-ups) and entrepreneurship. Specifically, it focuses on the barriers and challenges that SMEs face in regard to growth, the transition to more sustainable business models and digitalization (European Commission 2020).

Specifically, to measure innovation, the question “During the past 12 months, has your enterprise introduced any of the following types of innovation?” is used. This question has various possible answers. In our empirical analysis, the following items are used: “A new or significantly improved product or service to the market” is used to measure product innovation (fsINPROD), and “A new or significantly improved production process or method” is used to measure process innovation (fsINPROC).

On the other hand, to measure the barriers to digitalization (fsBAR) illustrated by the Flash Eurobarometer 486, the following question is chosen: Which of the following, if any, is a barrier to digitalization in your enterprise? The following two options are used: information technology security issues (fsSEC) and uncertainty regarding future digital standards (fsUNC). The variable “barriers to digitalization” (fsBAR) is obtained by calculating the joint effect of the two obstacles by means of the command “fuzzyand”. Tables 2 and 3 show that this variable is preceded by the symbol “ ~ ”. This symbol in fsQCA indicates the absence of the variable; therefore, in empirical analysis, we study how the absence of barriers to digitalization favours entrepreneurship. Moreover, the symbol “*” indicates the combination of variables that improve the outcome.

**Table 2:** Sufficient causal configurations to improve competitiveness

	Raw coverage	Unique coverage	Consistency
fsdigS*fsINPROD	0.718	0.220	0.904
fsTEA*fsINPROD*fsINPROC	0.503	0.005	0.707
Solution coverage: 0.724			
Solution consistency: 0.748

**Table 3:** Sufficient causal configurations to influence entrepreneurship

	Raw coverage	Unique coverage	Consistency
fsVC* $\sim$ fsBAR	0.529	0.267	0.771
fsVC*fsDIGS*fsITAL*	0.264	0.002	0.742
fsDIGS*fsITAL* $\sim$ fsBAR	0.377	0.115	0.745
Solution coverage: 0.647			
Solution consistency: 0.713			

The sample is composed of 19 European countries, and the selection of these countries was based on the availability of data in the different databases used. As mentioned above, this type of analysis is particularly suitable for small samples (N = 10–50 cases) (Ragin 2008b). The data regarding the variables used in this research are drawn from surveys that collected the information from 2019 until early 2020.

To calculate the causal configurations, the truth table algorithm is used (Ragin 2008b). The truth table treats each case as a combination of the selected characteristics (or “configuration” in fsQCA terminology) (Kent and Olsen 2008; Iannacci and Kraus 2022). Additionally, the truth table calculates all the possible 2 k combinations of potential causal conditions (where k is the number of causal conditions) and records both the number of cases featuring that configuration and whether the outcome happened (Pappas and Woodside 2021).

The calculation of the truth table offers three alternative results: complex, parsimonious, and intermediate solutions. The complex solution does not simplify sufficient configurations, and is therefore the solution that offers the most details. The parsimonious solution simplifies matters as much as possible based on the consideration that all counterfactual information leads to the outcome of interest. Finally, the intermediate solution assumes that only some of the possible causal configurations that do not pertain to actual cases have led to the results analysed by Ragin and Sonnet (2005). Ragin (2008a) recommends the use of the intermediate solution because it is usually the most interpretable. Additionally, to calculate the truth table, the analysis uses the Quine-McCluskey algorithm.

### Results and discussion

The proposed equations are estimated using fsQCA 3.0 software by means of truth table analysis. In addition, it is assumed that all the variables are present and, in accordance with the suggestion of Ragin (2008a), the intermediate solution is used.

Tables 2 and 3 show the results, include measures of coverage and consistency for each solution term and for the whole solution. Consistency (with sufficiency) measures the degree to which solution terms and the solution as a whole are subsets of the outcome. Coverage measures how much of the outcome is covered or explained by each solution term and by the solution as a whole (Ragin 2008b). In addition, solution coverage and solution consistency refer to the whole model. Instead, consistency and raw coverage refer to each causal configuration. Woodside (2018) emphasized the importance of achieving high consistency over high coverage. In the two equations estimated, the sufficient configurations exhibit consistencies higher than 0.7, indicating that the models collect most of the cases under study.

In Eq. 1, the global competitiveness variable (fsCOMP) is considered to be the outcome, and the explanatory variables that allow the causal configurations to be obtained are entrepreneurship (fsTEA), digitalization systems (fsDIGS), product innovation (fsINPROD) and process innovation (fsINPROC).

1 $${\text{fsCOMP}}{\mkern 1mu} { = }{\mkern 1mu} {\text{f}}\;\left( {{\text{fsTEA}},{\text{fsDIGS}},{\text{fsINPROD}},{\text{fsINPROC}}} \right)$$

Based on the calculation of the truth table of Eq. 1, two paths are obtained that have been followed by the countries under consideration (see Table 2). The first causal configuration, “fsDIGS*fsINPROD”, accounts for 71% of the cases (with a consistency of 0.904). This causal configuration indicates that the countries with the highest global competitiveness index are those that feature entrepreneurs who introduce product innovations as well as a framework of formal and informal institutions and socioeconomic conditions that favour the digitalization of entrepreneurial activity. Therefore, based on this causal configuration, research questions 1 and 2 can be answered.

Specifically, there is a positive relationship between product innovation and competitiveness. This result is in line with the findings of Kuratko and Audretsch (2009) and Petrakis et al. (2020) since entrepreneurs introduce product innovations in the attempt to obtain competitive advantages in the market, thereby ultimately increasing global competitiveness. Likewise, this causal configuration also allows question 2 to be answered; namely, countries that have favourable environments and physical, human, and financial resources for the digitalization of companies are more competitive (Kraus et al. 2018; Nobanee and Dilshad 2020; Schwab 2019; Autio et al. 2020).

The second path shown in Table 2 indicates that 50% of the cases opt for the following combination of variables, “fsTEA*fsINPROD*fsINPROC” (with a consistency of 0.707), thus allowing us to answer research questions Q1 and Q3. Therefore, the existence of greater entrepreneurial activity fosters national and economic competitiveness since entrepreneurs introduce both product innovations and process innovations, which promotes greater competitiveness. This combination of variables confirms the theses proposed by Schwab (2019) and Autio et al. (2020).

Equation 2 refers to entrepreneurship (fsTEA), and the variables that are considered to obtain the causal configurations are the absence of barriers to digitalization (~ fsBAR), venture capital (fsVC), digitalization systems (fsDIGS) and investment in talent (fsITAL).

2 $${\text{fsTEA}} = {\text{f}}{\mkern 1mu} (\sim {\text{fsBAR}},{\text{fsDIGS}},{\text{fsITAL}},{\text{fsVC}})$$

Table 3 shows the causal configurations that influence entrepreneurship. It can be seen that 53% of the cases follow this path: availability of venture capital and absence of barriers to digitalization fsVC* ~ fsBAR (with a consistency of 0.771). This causal configuration answers research question Q5. The main obstacles that prevent entrepreneurial activity from taking advantage of the business opportunities offered by digitalization include security problems, uncertainty regarding future digital standards, and the lack of financial resources (European Commission 2020). Thus, countries in which the barriers to digitalization are lower and entrepreneurs are able to find financing for their productive activities through venture capital funds exhibit higher rates of entrepreneurial activity. These results confirm the approaches of Ernst and Young (2015), Bessière et al. (2019), and Acs et al. (2020).

In addition, according to Table 3, two other paths can answer questions Q4 and Q5. These two causal configurations share two common explanatory variables of entrepreneurship– the digital environment (fsDIGS) and investment in talent (fsITAL) – but they differ in that 26% of cases combine the availability of venture capital with the previous elements (fsVC), while 38% of the cases combine it with the absence of barriers to digitalization (~ fsBAR).

Therefore, to take advantage of business opportunities that involve new digital technologies, it is essential to invest in talent. If investment in human capital is greater, entrepreneurs can introduce digital technologies and take advantage of the changes that digitalization entails (Autio et al. 2020), thus creating new ventures. In addition, entrepreneurs need a qualified workforce to introduce innovations into the market (Rippa and Secundo 2019; Dorasamy 2021); therefore, Q4 can be answered in the affirmative, i.e., investment in talent positively affects entrepreneurship.

## Implications

The empirical analysis thus conducted illustrates how innovation can have a positive effect on competitiveness since entrepreneurship can improve the efficiency of companies’ production processes and enable them to generate cheaper and better quality goods and services, which allows them to access a greater number of markets and satisfy the needs of more potential consumers.

In this sense, it is necessary to consider the advantages offered by a digitalization environment in this process, since it allows entrepreneurs to obtain more information and greater knowledge of both the labour market and the markets that it intends to access with its production; such an environment also allows workers to improve their skills, resulting in higher productivity.

For this reason, it is necessary to design policies to encourage this process. On the one hand, tax incentives that offer deductions for the implementation of this type of investment can have a positive effect. However, in terms of spending, subsidies could be provided to lower the costs of companies´ digitalization process. These expenditures can have positive effects with regard to stimulating the digitalization process. First, this approach could ensure the financing of research and development centres for digital processes, the results of which can be applied to production processes. Second, it could generate expenditure dedicated to improving human capital, which fulfils a dual purpose: on the one hand, it enables people to assimilate and take advantage of the innovations that are generated, thus improving their skills and productivity, while on the other hand, it promotes talent, which facilitates not only the implementation of the innovations generated but also the creation of new processes. In this case, such talent could be used by the research and development centres thus created.

In this context, it is important to highlight the importance of an adequate design of educational policy that can allow human capital to take advantage of the digitalization process, especially the design of university study plans for this purpose. In this sense, the relationship between universities and companies is especially relevant when designing these study plans and implementing innovations that are suitable for improving competitiveness.

Finally, alongside these actions, we must add the implementation of a credit policy that facilitates the capture of adequate financial resources to finance these innovation processes and their implementation. Governments can implement different measures to facilitate access to capital for innovative start-ups through the provision of funds and the financing of venture capital activities. This support can take two forms: financing existing venture capital funds and structuring public funds (Keuschnigg and Nielsen 2003; Berger and Hottenrott 2021). In addition, since the 2008 crisis, the trend of venture capital companies financing innovative projects related to the digitalization of companies and/or digital entrepreneurship has been increasing (Ernst and Young 2015; Daniels et al. 2016; Bèssiere et al. 2019; Acs et al. 2020); accordingly, policies must be designed to protect property rights and facilitate the emergence of more venture capital companies.

## Conclusions

The unstable economic situation resulting from the effects of the pandemic has led to a reduction in the economic growth of various countries. As a result of the vaccination process, the negative effects of the pandemic on health have been reduced, which has led to a higher level of activity and thus an improvement in growth; however, in many cases, the economic levels that had been reached prior to the pandemic have not been regained due to the negative effect of the pandemic on employment and well-being.

To improve this situation, one possibility that should be considered pertains to improving the competitiveness of companies to enable them to access more markets and meet greater possible demands. In this process, we have focused on digitalization and entrepreneurship, two factors that can positively affect competitiveness, since, on the one hand, entrepreneurs introduce into their production process those ideas and procedures that allow their companies to offer higher-quality goods and services, and, on the other hand, digitalization promotes the growth of production in all sectors of the economy and improves the efficiency of industrial processes, thereby fostering competitiveness and favouring entrepreneurial activity.

To analyse the relationships among these three variables considered together, an empirical analysis was conducted using fsQCA, which allowed us to find different combinations of variables that stimulate competitiveness and entrepreneurship. The main conclusions that can be drawn from the empirical analysis are as follows. First, countries with the highest competitiveness feature entrepreneurs who introduce product innovations and/or improve their production systems, thereby offering higher quality products and/or at lower costs to their clients. In addition, these countries also exhibit environments and resources that facilitate the digitalization of companies, which has a positive influence on competitiveness.

Second, according to the empirical analysis, entrepreneurs can take advantage of the business opportunities offered by digitalization if there are no network security problems or uncertainty regarding future digital standards and, moreover, if they have access to financial resources provided by venture capital firms. On the other hand, it can also be concluded that the stimulation of entrepreneurial activity requires environments that are favourable to digitalization as well as a greater investment in talent that allows the advances that digital technologies entail to be assimilated and utilized.

The implications of this type of study can be specified in the actions that should be designed when promoting the digitalization process in economies and thus take advantage of the business opportunities that have arisen as the digitalization process of companies has accelerated since the pandemic. Measures must be designed not only to improve the training of human capital so that it can assimilate the digitalization process and thus take advantage of the advantages that this entails in the tasks it performs, but also to favour the introduction of innovations in said process. Likewise, measures must be introduced to promote and guarantee the financing of this process, without which it is difficult for some sectors to implement this digitalization process.

The main limitation of the study is the low availability of data in the case of some of the variables considered in the empirical study for recent years. This implies that not all of the changes that have occurred during the pandemic can be observed. In this sense, one of the additional problems that we find is that these data are not calculated for large samples of countries, so the sample to be considered is not as large as would have been desirable. Alongside this, it must be added that it is not possible to carry out an analysis of a group of countries, to see the structural differences, due to the lack of data scarcity for some of them, which can be carried out when said data are published, considering the effects that the recent pandemic has had on the relationships between the variables considered in this paper, also considering different groups of countries, and it can also be differentiated by the motivations for entrepreneurship (taking advantage of a business opportunity, lack of job opportunities, continuing with a family business).

## Data Availability

The data that support the findings of this study are available from the corresponding author upon request.
